# Correction: Hui et al. Wrangling Actin Assemblies: Actin Ring Dynamics during Cell Wound Repair. *Cells* 2022, *11*, 2777

**DOI:** 10.3390/cells12111532

**Published:** 2023-06-02

**Authors:** Justin Hui, Viktor Stjepić, Mitsutoshi Nakamura, Susan M. Parkhurst

**Affiliations:** Basic Sciences Division, Fred Hutchinson Cancer Center, Seattle, WA 98109, USA; jhui@fredhutch.org (J.H.); vstjepic@fredhutch.org (V.S.); mnakamur@fredhutch.org (M.N.)

In the original publication [1], there was a mistake in the legend for Figure 4. The correction of a typo was missed during the revision phase.

The correct legend of Figure 4 is as follows:

**Figure 4.** Branched and linear nucleation factors are crucial for cell wound repair. (**A**) Schematic diagram depicting crucial factors for stabilizing and nucleating linear actin filaments. Formins add actin monomers to the barbed end of the actin filament, whereas crosslinkers aid in bundling linear filaments. (**B**) Schematic diagram depicting branched filament assembly via the Arp2/3 complex and WAS family proteins. (**C**–**F**) XY super-resolution view of actin ring organization at 70% percent wound closure in control cell wounds showing a dense actin mesh circumscribing the wound (**C**), Diaphanous RNAi knockdowns (disrupting linear actin formation) exhibit a diffuse mesh of branched actin at the wound periphery (**D**), alpha-actinin RNAi knockdowns (an actin crosslinker needed for actin bundling) form a more sparse ring at the wound edge compared to that at control wounds (**E**), and Arp 2/3 RNAi knockdowns (disrupting branched actin formation) do not form an actin ring at the wound periphery, but rather have unusually long linear actin filaments within the wound (**F**). Scale bar: 5 µm. (**G**) Schematic diagram depicting different types of actin architectures. Reprinted from Ennomani et al., (2016), Curr. Biol. 26(5): 616–626. doi:10.1016/j.cub.2015.12.069 [60], with permission from Elsevier.

The authors state that the scientific conclusions are unaffected. This correction was approved by the Academic Editor. The original publication has also been updated.
